# Draft Genome Sequence of *Pseudomonas* sp. EpS/L25, Isolated from the Medicinal Plant *Echinacea purpurea* and Able To Synthesize Antimicrobial Compounds

**DOI:** 10.1128/genomeA.00346-16

**Published:** 2016-05-05

**Authors:** Luana Presta, Emanuele Bosi, Marco Fondi, Isabel Maida, Elena Perrin, Elisangela Miceli, Valentina Maggini, Patrizia Bogani, Fabio Firenzuoli, Vincenzo Di Pilato, Gian Maria Rossolini, Alessio Mengoni, Renato Fani

**Affiliations:** a Department of Biology, University of Florence, Florence, Italy; b Center for Integrative Medicine, Careggi University Hospital, University of Florence, Florence, Italy; c Department of Surgery and Translational Medicine, University of Florence, Florence, Italy; d Department of Medical Biotechnologies, University of Siena, Siena, Italy; e Department of Experimental and Clinical Medicine, University of Florence, Florence, Italy; f Clinical Microbiology and Virology Unit, Careggi University Hospital, Florence, Italy; g Don Carlo Gnocchi Foundation, Florence, Italy

## Abstract

We announce here the draft genome sequence of *Pseudomonas* sp. strain EpS/L25, isolated from the stem/leaves of the medicinal plant *Echinacea purpurea*. This genome will allow for comparative genomics in order to identify genes associated with the production of bioactive compounds and antibiotic resistance.

## GENOME ANNOUNCEMENT

The genus *Pseudomonas* consists of a group of bacteria particularly relevant from both medical and biotechnological viewpoints (1). Thanks to their metabolic versatility, they successfully colonized several different niches, including water, soil, plants, and animals. Here, we present the draft genome sequence of *Pseudomonas* sp. EpS/L25, a strain close to *Pseudomonas oleovorans*, isolated from the stem/leaves of *Echinacea purpurea*, a medicinal plant whose essential oil possesses antimicrobial activity (2). The *E. purpurea* plants were collected in October 2012 (3) at the “Giardino delle Erbe,” Casola Valsenio. Medicinal plants are known for their beneficial effects for humans (including their antibacterial activity), but, in spite of their high relevance, endophytic bacterial communities inhabiting their rhizosphere or internal tissues are almost totally unknown. Thus, it is still unknown if they contribute to the antimicrobial activity exerted by *E. purpurea* extracts.

Previous characterization of *Pseudomonas* sp. EpS/L25 revealed the ability of this strain to inhibit the growth of other *E. purpurea*-associated bacteria (4) and, more interestingly, some opportunistic bacterial pathogens belonging to the *Burkholderia cepacia* complex. Furthermore, it showed resistance to several antibiotic compounds (5). Due to these properties, it represents a good candidate for further molecular investigations on the genetic basis of such features, prompting for sequencing of its genome.

The genome sequence of *Pseudomonas* sp. EpS/L25 was determined by a 2 × 300-bp paired-end approach using the MiSeq sequencing system (Illumina Inc., San Diego, CA, USA). A total of 3,020,786 paired-end reads were obtained, representing approximately 158× coverage of the whole genome. *De novo* assembly was performed using SPAdes version 3.5 (6), which generated 300 contigs. Contigs with length less than 2,000 bp were discarded and the remaining ones used for a multi-draft-based analysis using 16 *Pseudomonas* genomes retrieved from the NCBI database (*Pseudomonas* ND6, *Pseudomonas* TKP, *Pseudomonas* VLB120, *P. aeruginosa* B136 33, *P. aeruginosa* UCBPP PA14, *P. brassicacearum* NFM421, *P. denitrificans* ATCC 13867, *P. entomophila* L48, *P. fluorescens* R124, *P. mendocina* NK 01, *P. poae* RE 1 1 14, *P. putida* BIRD 1, *P. putida* KT2440, *P. stutzeri* CCUG 29243, *P. syringae* B728a) through MeDuSa scaffolder (7). The final version of the genome embeds 18 scaffolds, the longest of which is 1,664,566 bp long. The draft genome assembly of *Pseudomonas* sp. EpS/L25 has a total length of 5,435,234 bp. The G+C content is 65.5%, similar to that of other *Pseudomonas* genomes. Automated annotation of the *Pseudomonas* sp. EpS/L25 draft genome sequence using NCBI Prokaryotic Genome Annotation Pipeline detected 4,690 protein coding genes, 76 RNA coding genes (5 complete rRNAs, 57 tRNAs, 14 ncRNAs), and 105 pseudogenes. Three CRISPR arrays were also identified.

Comparative genomics analysis confirmed the presence of antibiotic efflux pumps, some conferring specific resistance to beta-lactams (*pdc*), florfenicol (*cfrA*), and polymyxins (*arnA* and *pmrF*)*.* Moreover, genes involved in the production of secondary metabolites with antimicrobial activity have also been detected (terpene, aryl-polyene, and two nonribosomal peptides).

### Nucleotide sequence accession numbers.

This whole-genome shotgun project has been deposited at GenBank under the accession number LNUP00000000. The version described in this paper is the first version, LNUP01000000.
